# An Authentic Inner Compass and Need Satisfaction as Wellbeing Resources in Bedouin Teaching Students During the COVID-19

**DOI:** 10.3389/fpsyt.2022.870764

**Published:** 2022-07-07

**Authors:** Rinat Cohen, Ortal Slobodin

**Affiliations:** ^1^Baruch Ivcher School of Psychology, Interdisciplinary Center (IDC), Herzliya, Israel; ^2^Department of Education, Achva Academic College, Arugot, Israel; ^3^The Department of Education, Ben-Gurion University, Beer-Sheva, Israel

**Keywords:** authentic inner compass, Bedouin, COVID-19, higher education, Self-Determination Theory

## Abstract

A growing body of literature suggests that students from underserved backgrounds are more vulnerable to the adverse economic, emotional, and academic effects of the current COVID-19 pandemic. While this vulnerability was attributed to multiple structural and socio-cultural barriers, little attention has been paid to the role of psychological resources in preserving wellbeing in times of crisis and change. Guided by the Self-Determination Theory (SDT), the current study examined the role of the authentic inner compass (AIC) and need-satisfaction in predicting the wellbeing of Bedouin students attending teachers' higher education institutes in the south of Israel during the COVID-19. Participants were 84 Bedouin teaching students (84.1% female) who completed online questionnaires addressing the sense of AIC, need-based experiences, psychological distress, and positive affect. Consistent with the propositions of the SDT, we found that a strong and clear sense of AIC, as well as high need satisfaction and low need frustration, were associated with lower distress and higher positive effect in Bedouin teaching students. We have also found that need satisfaction moderated the effect of the AIC on students' wellbeing so that AIC better predicted lower distress and higher positive effect when students' levels of need satisfaction were higher. Our findings lend further support to the importance of the AIC and need satisfaction to optimal functioning even in collectivist cultural contexts that do not prioritize values of autonomy. The current study provides insight into the interplay between AIC and need-based experience by describing the conditions under which AIC may be beneficial for wellbeing in times of crisis.

## Introduction

The emergence of Corona Virus disease (COVID-19) has forced educational institutions to close their gates and move from traditional face-to-face learning to distance education almost overnight. While this rapid transition has been accompanied by pedagogical, technological, and emotional difficulties for most students (1, 2), these challenges were exacerbated for those from underserved populations (3, 4). Previous studies conducted before and during the COVID-19 have shown that students from underserved backgrounds (e.g., such as culturally and linguistically minority students, students with low socioeconomic status, students living in rural areas, and first-generation to higher education students) encountered multiple structural and socio-cultural challenges related to distance learning. These difficulties included the lack of access to technological resources (5), limited time and space (6), disconnection from faculty members and peers (7), lack of culturally relevant pedagogies (8, 9), and difficulties in adjusting to self-directed learning (10).

In light of the disproportional adverse impact of the COVID-19 on the educational and psychological outcomes of students from underserved populations (11), recent studies began to examine potential resources that might buffer these harmful effects (12, 13).

Guided by the Self Determination Theory (SDT), the current study focused on the authentic inner compass (AIC) (14) and need satisfaction as two potential psychological resources that may contribute to the wellbeing of Bedouin students attending teachers' higher education institutes in the South of Israel during the COVID-19.

The term “authentic inner compass” refers to inner guiding schemas that inform us on what is truly important for us and help us direct our behaviors and future decision-making. Given that both AIC and need satisfaction were associated with adolescents' and young adults' self-esteem, mental health, and adaptive coping (15–18), we examined how having a sense of an AIC and need-based experiences (satisfaction or frustration) are related to Bedouin minority students' psychological distress and positive affect during the COVID-19. Furthermore, we examined whether students' need-based experiences moderate the association between their sense of AIC and the levels of psychological distress and positive affect.

Understanding the role of AIC and need-satisfaction in minority students' wellbeing may be of high empirical and practical significance to policymakers, academic institutes, faculty members, and teaching students themselves.

### Need-Based Experiences and Their Associations With Students' Wellbeing

The SDT is a general motivation theory that posits that people are inherently prone toward psychological growth and integration, and thus toward learning, mastery, and connection with others (19, 20). SDT argues that for healthy development to unfold individuals require support for three basic psychological needs (21), namely those for autonomy (the feeling of being the origin of one's own behaviors), competence (feeling of achieving desired outcomes), and relatedness (the feeling of being understood and cared for by others). According to the SDT, the experience of need satisfaction serves as an internal resource of motivation and provides energy for exploration and growth (20) across cultures (22, 23) and life circumstances (24, 25).

Studies conducted among school and higher education students have shown that the satisfaction of the three basic psychological needs was associated with beneficial health, emotional and education outcomes, such as agentic engagement (26), autonomous motivation (27), prosocial behavior (28), and life skills development (29). In contrast, the frustration of the three basic psychological needs was associated with maladaptive outcomes, including lower students' engagement (30), decreased autonomous motivation (31), lower prosocial behavior (28), and higher academic drop-out rates (32). These conclusions appear to hold irrespective of whether researchers used a total score of need satisfaction encompassing the three psychological needs [e.g., (33, 34)], or on distinct measures of the three needs [e.g., (35, 36)].

While most studies within the SDT framework involved children and adolescents, understanding need satisfaction may be particularly important in the higher education context, given the self-determined nature of this education (especially during distance learning). Indeed, Gillet et al. (37) who studied changes in need satisfaction in the course of the first university semester found that students with moderate and increasing levels of need satisfaction reported higher levels of positive affect and effort, while students with low and decreasing levels of need satisfaction reported lower levels of positive affect, effort, and achievement, and higher levels of negative affect.

In times of crisis and change, need satisfaction may act as a resilience factor, attenuating the adverse impacts of the situation on individuals' wellbeing (38). Weinstein and Ryan [(39), p. 12] proposed that need satisfaction “buffer in times of stress, reducing both initial appraisals of stress and encouraging adaptive coping after stress-related events occur.” Thus, in the current study, we assumed that students' experience of need satisfaction would promote feelings of wellbeing and growth during the COVID-19 pandemic. In contrast, we assumed that students with high levels of need frustration would experience the COVID-19 as more challenging and difficult to cope with, and thus would exhibit higher distress.

### The Authentic Inner Compass as a Wellbeing Resource

An important aspect of the need for autonomy that is likely to become particularly significant in adolescence and emerging adulthood [e.g., (15, 16)], is a sense of having an AIC. Rooted mainly in SDT (20), and partly on Mill's (40) notion of liberty, Assor (14) proposed that the striving to self-organize and self-direct in ways that allow self-actualization and sense of volition is a core feature of the need for autonomy. When such guiding schemas exist, we feel that we have values, life aspirations, interests, and goals that function like an “authentic inner compass,” that informs us on what is truly important for us and help us direct our behaviors and future decision making. When we do not have such action- and decision- guiding inner compass, we are likely to feel confused and not capable of self-endorsed and volitional, self-direction, because we do not know what actions to choose (15, 16). Having a sense of AIC may be particularly important during late adolescence and emerging adulthood, when many central life decisions and identity commitments are made, particularly in societies offering a wide range of choices (17).

Past research on the AIC construct has shown that the experience of having an AIC was associated with a wide range of need satisfaction outcomes including vitality, low levels of depression, sense of meaning, life satisfaction, happiness, autonomous motivation to learn, resistance to negative pressure, tolerance for ambiguity, absence of attachment avoidance, clear and autonomous future plans and higher self-esteem (15, 16, 41–44). Although the AIC is likely to be of special importance in many post-modern, information-flooded, and moral relativistic societies, previous studies have demonstrated that the experience of having an AIC might be related to wellbeing also in hierarchical-collectivist cultures that traditionally do no emphasize authenticity and personal autonomy, such as Chinese and Bedouin (17, 44). For example, studies with Chinese showed that having a sense of AIC was related to growth promoting qualities such as self-congruence, intrinsic life-goals, and tolerance for ambiguity (41, 44) as well as with indicators of wellbeing, such as vitality, and self-esteem (17).

### Why Need-Based Experiences May Serve as Moderating Factors in the Association Between AIC and Students' Wellbeing?

While past research consistently shows that AIC contributes to many adaptive outcomes, the factors that promote or hinder the ability to translate this sense of self-directness into wellbeing and coping remained unclear. According to the SDT, need satisfaction is crucial to the development and maintenance of high-quality motivation and optimal functioning (19, 45). Hence, evaluating need satisfaction as a moderator in the AIC-wellbeing relationship could offer a better understanding of the psychological experiences that can promote students' adaptation.

Perceptions of autonomy, competence, and relatedness are fueled by socio-contextual factors in students' life. Previous studies within the SDT framework traditionally investigated basic need satisfaction as a mechanism (mediator) in the relationship between teachers' or parents' behavior and students' outcomes (26, 46). Yet, perceptions of autonomy, competence, and relatedness can be viewed as essential psychological factors that facilitate individuals' ability to use their AIC as a personal resource in stressful situations. Indeed, a few recent studies illustrated that need-based experiences played a moderating role in predicting wellbeing and distress. For example, Boudrias et al. (47) showed that need satisfaction moderated the relationship between job demands and turnover intention among nurses. Specifically, the study found that nurses who experience feelings of autonomy in their workplace were better equipped to deal with situations of role ambiguity and role conflict. In a similar vein, Kranabetter and Niessen (48) found that the satisfaction of the need for relatedness moderated the relationship between work appraisals and employees' depressive symptoms. They argued that employees who felt connected and secured found it easier to successfully integrate their work roles into their selves. In this case, appreciation, as behavior that addresses personal values as a genuine reward, might evoke positive emotions, thereby limiting depressive symptoms.

Together, these studies highlight the importance of investigating need-based experiences as factors promoting or hindering students' ability to translate the sense of AIC into wellbeing and functioning. Examining the moderating role of need experiences may be particularly important in times of crisis and change because emergencies, especially those eliminating social relationships, are inherently need suppressing (49, 50).

Thus, in the present study, we sought to extend the application of the AIC by considering need-based experiences as moderators of the AIC-wellbeing relationship. We argue that when need satisfaction is high, a sense of AIC would be experienced as a genuine resource for a behavior that is highly self-relevant and valued. It is in such a case that having a strong sense of AIC may particularly unfold its impact on positive emotions, making depressive and anxious symptoms less likely. However, having a sense of AIC may not be that beneficial for students' wellbeing when they feel unable to translate their personal goals into real-world behavior and achievements. For example, having a sense of AIC affects wellbeing more positively if a student feels free to make volitional choices regarding his or her life in general and in the academic context particularly, rather than when he or she feels incompetent, pressured, or controlled. When students perceive themselves as agents and consider their enactments more interesting, joyful, and meaningful (satisfaction of the need for autonomy), if the institute provides opportunities to develop skills and attain valued outcomes by mastering challenging tasks (satisfaction of the need of competence), and when students have a sense of belongingness and secure (satisfaction of the need for relatedness), then the association between having clear educational aspirations and plans would be associated with higher levels of wellbeing and functioning. In contrast, when students lack interest and choice in their studies (frustration of the need for autonomy), when they feel ineffective, bored, or over-challenged (frustration of the need for competence), or when they feel lonely and disconnected from faculty members and peers (frustration of the need for relatedness), having a clear identity and self-directed goals may not be enough to protect their wellbeing, particularly in times of crisis and change.

### Bedouin Students in the Israeli Higher Education System

The Bedouins are a unique subset of Israel's Arab population who number ~270,000 (about 13% of the total Israeli Arab population and 3% of Israel's entire population) (51). Bedouin families tend to be authoritarian, hierarchical, and oriented toward the tribal group. Fathers are the head of the family, and women, although educated, are expected to defer to husbands and fathers and to remain socially confined to the familial/tribal circles (52, 53). As a minority within a minority, the Bedouins have the lowest education level, below-average income per family, and the highest unemployment rates (54). Approximately half of the Bedouin community in the Negev lives in unrecognized settlements, most not connected to water or electricity and situated far from the main roads. Polygamy, although illegal in Israel, is prevalent among 30% of Bedouin women and is associated with increased levels of physical and psychological distress (55).

The number of Bedouin students enrolled in undergraduate programs in Israeli institutions has doubled over the last decade. However, these students still have lower enrollment rates, lower achievements, and higher dropout rates than their Jewish peers (56). These disparities were attributed to multiple barriers, including inadequate high-school preparation, language challenges, and financial difficulties (57). The Israeli Knesset (58) reported that about one-third of 17-year-old Negev Bedouins did not attend school in the 2015–2016 academic year, and only 32% of 18-year-old Bedouin received matriculation certificates. Accordingly, only about one-third of Bedouin candidates met entry requirements for universities, as opposed to 68 percent of the general population.

Similar to other students from disadvantaged backgrounds (3), Bedouin high education students faced many structural and cultural challenges as a result of the sudden shift to distance learning. A recent study focusing on Bedouin high education students found that 90% of the interviewed students (*N* = 257) experienced extreme difficulties in adjusting to distance learning during the COVID-19 pandemic and that more than half estimated their chances of dropping out to be moderate to high (59). A study that compared Bedouin and Jewish teaching students' experiences of the shift to distance learning during the COVID-19 has shown that Bedouin students' learning was hindered not only by the lack of digital resources but also by gender-based traditional values and roles (60). For example, while Jewish female students, who were often single and without children, found synchronous online-learning methods (real-time live lessons delivered through video conferencing platform) to be effective, Bedouin female students found them ineffective due to multiple domestic tasks and limited computers. Similarly, Manevitch-Malul et al. (61), who focused on the distance learning experiences of Israeli university students found that for many Bedouin students, the shift to distance learning increased the already existing feelings of disconnection and alienation from faculty members and peers and undermined their role as “students.” Moreover, Bedouin university students faced increased conflicts between their educational demands and family obligations, especially when their families could not understand or accept their need to continue studying even while staying at home.

### The Current Study

Previous studies on minority students have consistently pointed to ethnic and racial differences in higher education enrollment and performance (62, 63). These disparities were exacerbated by the current COVID-19 pandemic which exposed minority groups worldwide to increased levels of health, financial, social, and educational stressors (64–66).

Given the evidence on the difficulties of students from the underserved backgrounds during the COVID-19 and the shift to distance learning (67), the main purpose of the current study was to examine whether and how a sense of AIC and need-based experiences are associated with psychological wellbeing and distress in Bedouin teaching students. We were also interested in examining whether a sense of AIC interacts with students' level of need satisfaction and need frustration in predicting psychological distress and positive affect. We expected that the positive association between AIC and positive affect would be enhanced by high levels of need satisfaction and attenuated by high levels of need frustration. In contrast, we expected that the negative association between AIC and psychological distress would be further exacerbated by high levels of need frustration and attenuated by high levels of need satisfaction.

The study was guided by two main hypotheses:

H1: Bedouin students' sense of AIC and need satisfaction will be positively associated with positive affect and negatively associated with psychological distress. In contrast, need frustration will be negatively associated with positive affect and positively associated with psychological distress.
H2a: The association between students' AIC and their level of psychological distress will be moderated by need-based experiences. Specifically, the negative association between AIC and psychological distress would be stronger for students with higher levels of need frustration than for students with lower levels of need frustration. In contrast, the association between AIC and psychological distress would be weaker for students with higher levels of need satisfaction than for students with lower levels of need satisfaction.
H2b. The association between students' AIC and positive affect will be moderated by need-based experiences. Specifically, the positive association between AIC and positive affect would be stronger for students with higher levels of need satisfaction than for students with lower levels of need satisfaction. In contrast, the association between AIC and positive affect would be stronger for students with lower levels of need frustration than for students with higher levels of need frustration.

## Methods

### Participants and Procedure

According to the Central Bureau of Statistics (56), there are 2,981 Bedouin students studying for a bachelor's degree in all Israeli higher education institutes (1.5% of the total student population in Israel). About half of the Bedouin students (53.2%) are studying education or teaching.

The current study included 84 Bedouin teaching students (84.1% female), studying in one of three higher education institutes that provide teaching training in the South of Israel (one university and two academic colleges). According to representative staff of these three institutes, the total number of Bedouin teaching students is estimated to be around 400 and 500 in a given time point. The vast majority of Bedouin teaching students learn in an academic college for education, which specializes in training pre-service teachers and includes a high proportion of Bedouin students (55%). Given this estimation, our sample size represents 17–20% of the total population of interest.

Participants' age ranged between 20 and 48 (Mean = 32.91, SD = 7.13). Most participants (69.5%) were parents with a mean number of 3.41 children per family (SD = 2.31). The background variables are presented in Table 1.

**Table 1:** Participants' background variables (*N* = 84).

	***N***	**Percentage**
**Gender**		
Male	13	15.9
Female	69	84.1
**Marital status**		
Single	21	25.6
Married	61	74.4
**Children**		
Yes	57	69.5
No	25	30.5
**Religious affiliation**		
Traditional	29	35.8
Religious	53	64.2
	**M (SD)**	**Range**
Number of children	3.41 (2.31)	0–13
Age	32.91 (7.13)	20–48

Data were collected between May and July 2021, while most Israeli academic institutes used distance learning methods. Participants were invited to participate in an online survey *via* Qualtrics. The survey was distributed through social media groups of Bedouin teaching students. Students were informed that their answers would remain anonymous and confidential. Participation was voluntary without remuneration. The research was approved by the institutional ethics committee.

### Measures

***Background variables*** included students' age, gender, marital status, number of children, and level of religiosity.

***Need satisfaction and frustration*** were assessed by the Basic Psychological Needs Scale (BPNS) (68). Participants filled out the full 24-item version that has 12 items tapping needs satisfaction, and 12 items tapping needs frustration. All items were rated on a 5-point scale ranging from 1 (strongly disagree) to 5 (strongly agree). Each 12-item scale has four items tapping autonomy, four items tapping competence, and four items tapping relatedness. For each participant, the total needs satisfaction and the total need frustration scores were calculated by taking the means of the 12 needs satisfaction scores, and the 12 need frustration scores, respectively. The BPNS has proven good validity, internal consistency, and temporal stability for each of the three factors (68). For example, structural equation modeling showed that autonomy, competence, and relatedness need satisfaction positively predicted active commuting to and from school (69). Previous studies supported the validity and reliability of the BPNS across contexts and cultures (70, 71), with internal consistency ranging between 0.64 and 0.89. Studies that used the scale was in students, beginning teachers, and pre-service teachers from the Bedouin society reported internal consistencies of 0.73–0.78 (72–74). In the current study, Cronbach alpha coefficient for need satisfaction was 0.91 and for need frustration 0.82.

***A sense of an authentic inner compass*** was assessed by the Authentic Inner Compass Scale (41). The scale consists of 11 items on a 5-point Likert-type scale ranging from 1 (not at all) to 5 (to a very large extent), including “I have goals that are personally important to me and I fully identify with,” and “I have values that truly reflect the kind of person I want to be.” The items were averaged to produce a total AIC score. Studies that examined the incremental and discriminant validity of the AIC scale showed that the AIC has weaker correlations with indicators of exploration and purpose-searching than with identification with commitment or identified purpose, supporting the distinctiveness of the behavioral self-realization and AIC concepts (15, 17). The AIC scale has also good criterion validity, showing strong positive correlations with freedom, volition, vitality, and negative correlation with depression (17).

A study that examined the validity of the scale in Bedouin adolescents reported an internal consistency of 0.63 (15). In this study, Cronbach's alpha coefficient was 0.92.

***Psychological distress*** was measured by Depression, Anxiety, and Stress Scales (DASS-21) (75). This widely used scale addresses three groups of symptoms: depression, anxiety, and stress, with seven items for each group. Participants responded on a 4-point Likert scale ranging from 0 (did not apply to me at all) to 3 (applied to me very much or most of the time). Previous studies supported the validity and reliability of the DASS-21 in a variety of cultural contexts (76, 77). In a non-clinical sample of American adults, Sinclair et al. (78) reported on good internal consistency (0.91, 0.80, and 0.84 for Depression, Anxiety, and Stress scales, respectively) and scale-level correlations (between 0.68 and 0.73). A recent study that used the DAAS-21 in Arab adults in Israel reported internal consistencies of 0.91, 0.87, and 0.90 for the Depression, Anxiety, and Stress scales, respectively (79). In the current study, the Cronbach's alpha coefficient for depression scale was 0.87, the anxiety scale was 0.90 and the stress scale was 0.86. The Cronbach's alpha coefficient for the total scale was 0.95.

***Positive affect*** was assessed by the Positive and Negative Affect Schedule (PANAS) (80). The 10 items in the positive affect scale reflect the extent to which a person feels active, enthusiastic, and alert. High positive affect is a state of high energy, concentration, and experiencing pleasure, whereas low positive affect is characterized by sadness and lethargy (80). Respondents are asked to rate the extent to which they have experienced each particular emotion within the past week, with reference to a 5-point Likert-type scale ranging from 1 (very slightly) to 5 (extremely). The high extreme of each dimension indicates a strong experience of affect, while the low end represents a weak experience of affect (81). The positive affect scale has good internal consistency, with Cronbach's alpha ≥0.84 across multiple time frames. The scale also demonstrates good convergent and discriminant validity (82). The scale was used in various cultural setting, showing good psychometric properties (83, 84). A study that examined the cross-cultural adaptation of the negative affect scale in Bedouin children reported an internal consistency of 0.77 (85). In Agbaria (86, 87) who studied Arab adolescents in Israel, internal consistencies of α = 0.80 for the positive affect scale and 0.79 for the negative scale were found. In the current study, the Cronbach's alpha coefficient for the positive affect scale was 0.82.

### Confirmatory Factor Analysis

To provide validity information on the internal structure of the employed measurements in the current sample, we conducted a confirmatory factor analysis (Appendix 1). This analysis examined the convergence of the items into factors. The measurement model was composed of 15 manifested items pertaining to the five study variables: need satisfaction, need frustration, authentic inner compass, positive affect, and psychological distress. All the latent variables were assessed by three parcels of items. Variables items were randomized into one of the three parcels. We used parcels to create a reasonable ratio of observed indicators with respect to the sample size (88, 89). For the DAAS-21 scale, factorial construct validity was conducted using the three-factor model, including depression, anxiety and stress. The results indicated an adequate fit to the data, $\chi_{\left(80\right)}^{2}$ = 115.51, *p* = 0.006, CFI = 0.96, RMSEA = 0.07. Parcel loadings onto their respective factors were all strong and statistically significant, ranging from 0.40 to 0.98, which validated the measurement model.

### Data Analyses

To test H1, bivariate Pearson correlations between study variables were calculated. To assess H2, conditional process modeling was used to test the moderating effect of need-based experiences on the association between AIC and students' wellbeing, as outlined by Hayes (90), using the PROCESS macro. We used an alpha level of 0.05 for all statistical tests. Data analyses were carried out on SPSS Windows 26.0.

Power calculations using G^*^ POWER calculator (91) revealed that for multiple regression with five predictors, alpha of 0.05 and effect size of 0.95, a minimum of 73 participants was required.

## Results

Table 2 presents the correlations between the participants' background variables and the study variables. Age was negatively associated with psychological distress. No other significant associations were identified.

**Table 2:** Correlations between socio-demographic and study variables.

	**Need**	**Need**	**AIC**	**Positive**	**Psychological**
	**satisfaction**	**frustration**		**affect**	**distress**
Gender	0.13	0.05	0.21	−0.04	−0.04
Having children	−0.01	−0.03	−0.01	−0.06	0.07
Religion	0.09	0.26	−0.01	−0.08	0.16
Marital status	−0.01	0.08	−0.09	−0.07	0.10
Age	0.17	−0.22	0.13	0.17	−0.39**
Number of children	−0.06	−0.06	−0.001	−0.01	−0.21

* *p < 0.05*,

** *p < 0.01*,

*** *p < 0.001*.

*This table presents the correlations between participants' background variables and the study variables. Age was negatively associated with psychological distress. No other significant associations were identified*.

Means, standard deviations, and correlations between the study variables are presented in Table 3. In line with H1, the results of the correlation matrix showed that AIC was positively associated with need satisfaction and positive affect. In contrast, AIC was negatively associated with need frustration and psychological distress.

**Table 3:** Descriptive statistics and correlations between study variables.

	**M (SD)**	**1**	**2**	**3**	**4**	**5**
1. Needs satisfaction	3.69 (0.74)	1				
2. Needs frustration	2.54 (0.68)	−0.14	1			
3. AIC	5.22 (1.14)	0.64**	−0.46**	1		
4. Positive affect	3.46 (0.70)	0.47**	−0.47**	0.71**	1	
5. Psychological distress	2.06 (0.76)	−0.43**	0.54**	−0.60**	−0.44**	1

* *p < 0.05*,

** *p < 0.01*,

*** *p < 0.001*.

*AIC, Authentic inner compass.*

*This table shows that AIC was positively associated with need satisfaction and positive affect. In contrast, AIC was negatively associated with need frustration and psychological distress*.

### The Interaction Effect of AIC and Need-Based Experiences on Students' Psychological Distress

**Figure 1:**
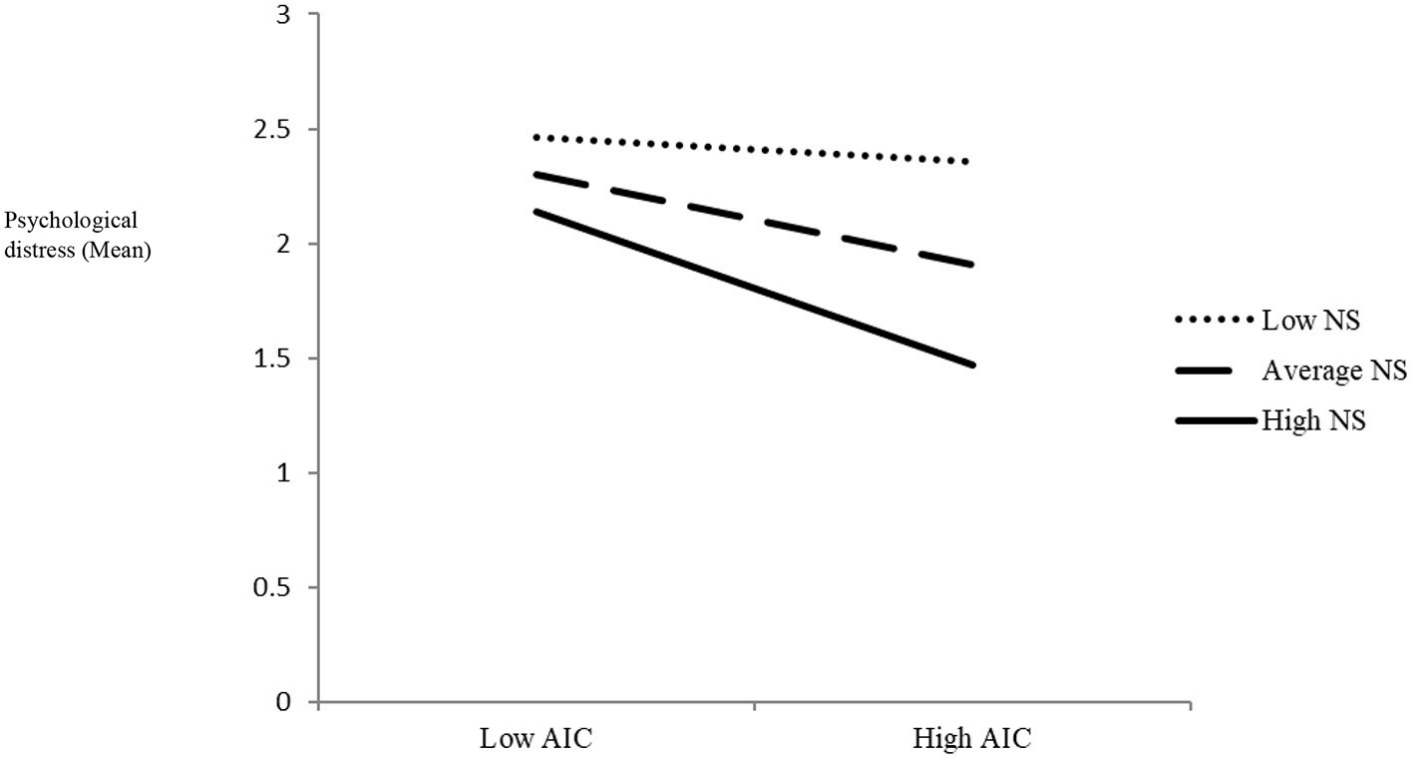
Interaction effect between AIC and need satisfaction (NS) on psychological distress. This figure displays the interaction plot for the association between AIC and psychological distress under the condition of low (−1 SD), medium (0 SD), and high (+1 SD) need satisfaction. As seen, when students' levels of need satisfaction were moderate or high, a stronger sense of AIC predicted lower psychological distress.

As seen in Table 4, results showed that AIC and need satisfaction had main effects on psychological distress (*B* = −0.11, *SE* = 0.05, *p* = 0.02 and *B* = −0.41, *SE* = 0.13, *p* = 0.001, respectively). Consistent with hypothesis H2a, the product term of the interaction between AIC and need satisfaction was significant (*B* = −0.11, *SE* = 0.05*, p* = 0.002), indicating that need satisfaction moderated the link between AIC and psychological distress. Simple slope analyses of the interaction effect showed that the relationship between AIC and psychological distress was significant under moderate (*B* = −0.11, *SE* = 0.05, *t* = −2.23, *p* = 0.02) or high (*B* = −0.20, *SE* = 0.06, *t* = −3.31, *p* = 0.001) levels of need satisfaction. That is, when students' levels of need satisfaction were moderate or high, a stronger sense of AIC predicted lower psychological distress. However, under low levels of need satisfaction, the link between AIC and psychological distress was not significant (*B* = −0.02, *SE* = 0.06, *t* = −0.42, *p* = 0.67). Figure 1 displays the interaction plot for the association between AIC and psychological distress under the condition of low (−1 SD), medium (0 SD), and high (+1 SD) need satisfaction.

**Table 4:** Regression analyses predicting Bedouin students' psychological distress and positive affect.

	**Psychological distress**					**Positive affect**				
	**β**	**B**	**SE**	**t**	***p***	**β**	**B**	**SE**	**t**	***p***
AIC	−0.26	−0.11	0.05	−2.23	0.02	0.55	0.22	0.05	4.62	0.001
Need satisfaction	−0.40	−0.41	0.13	−3.24	0.001	0.17	0.17	0.12	1.32	0.19
AIC* need satisfaction	−0.19	−0.11	0.05	−2.22	0.02	0.19	0.09	0.04	2.03	0.04
AIC	−0.38	−0.17	0.04	−3.93	0.001	0.53	0.21	0.04	5.19	0.001
Need frustration	0.39	0.42	0.10	3.87	0.001	−0.28	−0.29	0.10	−2.72	0.01
AIC* need frustration	0.21	0.13	0.05	2.54	0.01	0.02	0.01	0.05	0.29	0.77

*AIC, Authentic inner compass.*

*This table shows that need satisfaction moderated the link between AIC and psychological distress as well as the link between AIC and positive affect. Need frustration moderated the link between AIC and psychological distress*.

**Figure 2:**
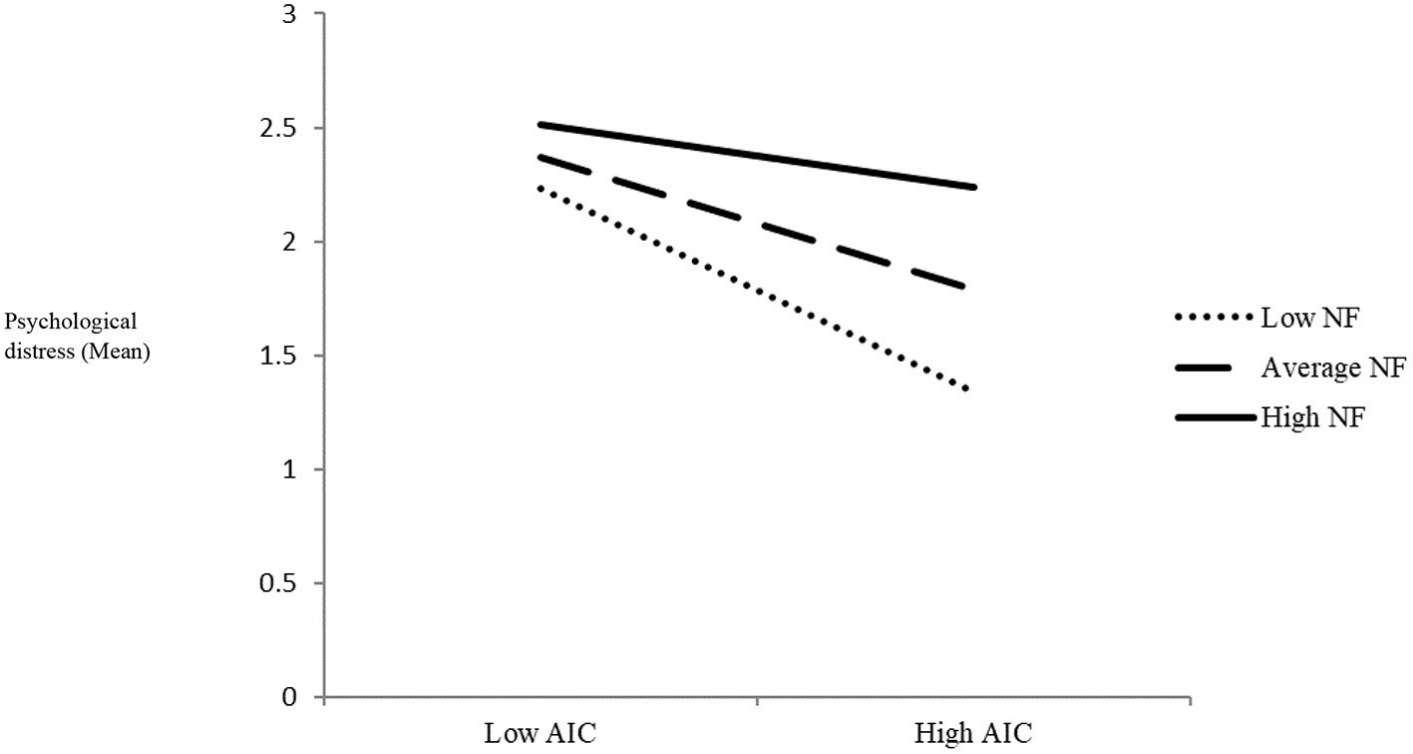
Interaction effect between AIC and need frustration (NF) on psychological distress. This figure displays the interaction plot for the association between AIC and psychological distress under low, moderate, and high levels of need frustration. As seen, when students experienced low or moderate levels of need frustration, a stronger sense of AIC predicted lower levels of psychological distress.

Testing the role of need frustration in the relationship between AIC and students' level of psychological distress showed that AIC and need frustration had main effects on psychological distress (*B* = – 0.17, *SE* = 0.04, *p* = 0.001, and *B* = 0.42, *SE* = 0.11, *p* = 0.001, respectively). Consistent with hypothesis H2a, the product term of the interaction between AIC and need frustration was significant (*B* = 0.13, *SE* = 0.05*, p* = 0.01), indicating that need frustration moderated the link between AIC and psychological distress. Simple slopes analyses indicated that AIC was significantly associated with psychological distress under low (*B* = −0.26, *SE* = 0.06, *t* = −4.43, *p* < 0.001) and moderate (*B* = −0.17, *SE* = 0.04, *t* = −3.93, *p* = 0.001) levels of need frustration but not under high levels of need frustration (*B* = −0.07, *SE* = 0.05, *t* = −1.47, *p* = 0.14). Accordingly, when students experienced low or moderate levels of need frustration, a stronger sense of AIC predicted lower levels of psychological distress. Figure 2 displays the interaction plot for the association between AIC and psychological distress under low, moderate, and high levels of need frustration.

### The Interaction Effect of AIC and Need-Experiences on Students' Positive Affect

**Figure 3:**
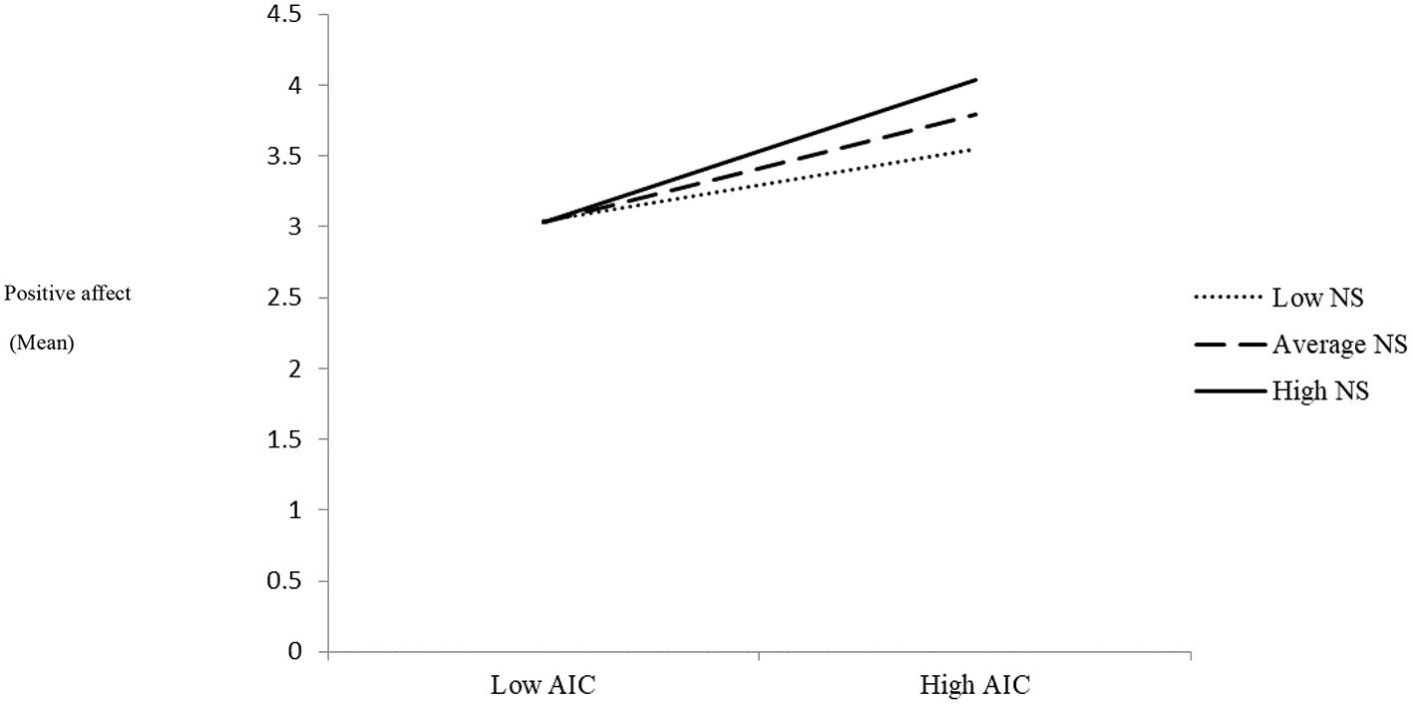
Interaction effect between AIC and need satisfaction (NS) on students' positive affect. This figure displays the interaction plot for the association between AIC and positive affect under low, moderate, and high levels of need satisfaction. As can be seen, the association between AIC and positive affect became stronger as the level of need satisfaction increased.

As seen in Table 4, AIC had a main effect on students' positive affect (*B* = 0.22, *SE* = 0.05, *p* = 0.001), while need satisfaction did not significantly predict the level of positive affect (*B* = 0.16, *SE* = 0.12, *p* = 0.19). Consistent with hypothesis H2b, the product term of interaction between AIC and need satisfaction was significant (*B* = 0.10, *SE* = 0.04*, p* = 0.04), indicating that need satisfaction moderated the link between AIC and positive affect. Simple slopes analyses indicated that the association between AIC and positive affect became stronger as the level of need satisfaction increased (low level of need satisfaction; *B* = 0.15, *SE* = 0= 0.06, *t* = 2.35, *p* = 0.02, moderate level; *B* = 0.22, *SE* = 0.04, *t* = 4.62, *p* < 0.001, and high level: *B* = 0.30, *SE* = 0.06, *t* = 5.21, *p* < 0.001). Figure 3 displays the interaction plot for the association between AIC and positive affect under low, moderate, and high levels of need satisfaction.

Testing the role of need frustration in the relationship between AIC and students' level of positive affect showed that AIC and need frustration had main effects on students' levels of positive affect (*B* = 0.21, *SE* = 0.04, *p* = 0.001 and *B* = −0.29, *SE* = 0.10, *p* = 0.008). However, the interaction effect between AIC and need frustration was not significant (*B* = 0.01, *SE* = 0.05, *p* = 0.77).

## Discussion

Our study aimed to investigate the roles of the AIC and need satisfaction in predicting the wellbeing of ethnic minority higher education students during the COVID-19. We applied the theoretical framework of SDT to explain how psychological distress (depression, anxiety, and stress) and positive affect could be predicted by AIC, need-based experiences (satisfaction or frustration), and their interaction.

Consistent with our hypotheses, we found that a strong and clear sense of AIC, as well as high need satisfaction and low need frustration, were associated with lower levels of distress and higher levels of positive affect among Bedouin teaching students. We have also found that need-based experiences moderated the link between AIC and Bedouin teaching students' psychological distress and positive affect. We also found that need frustration moderated the link between AIC and psychological distress.

Overall, our findings suggest that both AIC and need satisfaction may serve as motivating and energizing resources for minority students in times of crisis and change (92, 93). Our results align with the theoretical propositions of the SDT which state that individuals' psychological functioning derives not only from their environment but also from the psychological resources at their disposal (47). Support for the three basic psychological needs may be particularly essential in times of crisis, which are characterized by a continuous depletion of personal, economic, and social resources (94). Studies within the SDT framework suggested that the support of the three basic psychological needs may act as a resilience mechanism and facilitate adaptive coping strategies in times of crisis (38, 95). For example, Cantarero et al. (38) showed that higher levels of need satisfaction were related to adults' higher wellbeing during the COVID-19 outbreak and that intervention that supports the basic needs decreased perceived stress.

In addition to the positive association between need satisfaction and students' wellbeing, need satisfaction was also related to a stronger sense of AIC. This finding may be attributed to the energizing effects of need satisfaction on identity processes (96). Previous studies suggested that the support of the three basic psychological needs provides adolescents and adults essential resources and energy to explore existing identity options and facilitates greater self-organization and integrated identity development. Conversely, the frustration of psychological needs limits active and critical thinking and results in a fragmented, loosely integrated identity structure (97, 98). It is, therefore, possible that Bedouin students whose basic needs were satisfied had greater ability to intentionally seek out activities and contexts and make choices that are conducive to experiencing meaning and positivity. Probably, the relationship between need satisfaction and AIC is bidirectional, so that having a strong sense of identity, goals, and meaning increases the likelihood that students would experience their environment as need satisfying.

In line with previous studies that demonstrated the key role of the AIC in predicting adolescents' and young adults' self-esteem, mental health, and adaptive coping (15–18, 42), our findings suggest that having authentic values and goals contributes directly to students' wellbeing, as manifested in a higher level of positive affect and lower levels of distress.

Extending previous findings regarding workplace experiences (47, 48), we found that need-based experiences played a moderating role in students' wellbeing. Importantly, we found that the AIC may function as a wellbeing resource primarily in need-satisfying contexts. That is, students were able to enjoy the positive effects of the having an inner guideline only when they experience themselves as autonomous, related, and competent. However, when students' needs are thwarted (e.g., they feel controlled, incompetent in their abilities, or disconnected from campus life, faculty members, and peers) they may not be able to translate their AIC resources into wellbeing outcomes.

The role of the educational and social environments in supporting students' needs may be particularly crucial for Bedouin students, who are affiliated with a collectivist culture. Studies of culturally and linguistically diverse students in higher education emphasized the importance of family, peers, and institutional support in their adjustment, motivation, and performance (99, 100). This support includes aspects of pre-college socialization environments (school and home environment), financial assistance, balancing family and work responsibilities, and campus climates (e.g., racial/ ethnic discrimination) (101–103). Previous studies on minority students' reasons to pursue higher education showed that in addition to individual motivations (e.g., intellectual curiosity, personal interest, career aspirations), these students largely emphasized collective concerns, such as meeting family expectations and preserve community connections (104, 105). Nevertheless, strong connections with one's family and community may also have adverse effects on students' motivation and performance if they conflict with academic values and obligations (106). For example, Saenz and Ponjuan (107) noted that the tight solidarity in families of Latin origin may lead individuals to sacrifice their own needs for those of the family. Based on a study of 30 low-income Hmong American high school students, Lor (106) argued that poverty can create conditions in which family ties bind students to gender-based expectations and obligations that prevent them from pursuing opportunities for social mobility. These students are aware of their pivotal role in supporting their families and often frame family obligations as a significant barrier when it comes to achieving their own goals. Family obligations affect students in different ways. While males primarily framed cultural and religious obligations as time-consuming obstacles that interfere with their academic and social lives, females, who are expected to provide social and economic support, internalized guilt about being unable to fulfill their obligations in the future.

The collectivist characteristics of the Bedouin society and the traditional role teaching students (mostly female) play in their households may explain why family and community support are so essential for them in achieving their goals. When family and community connections are so strong and students are highly dependent on others, only environments that support students emotionally and financially and allow them to balance family obligations will allow them to follow their own values and benefit from having a clear and strong AIC.

Notably, although both need satisfaction and need frustration significantly moderated the effects of AIC on psychological distress, need frustration did not alter the relationship between AIC on positive affect. This finding may be explained by the SDT's differentiation between the “bright” pathway (where positive outcomes are more strongly related to need satisfaction) and a “dark” pathway (where negative outcomes are more strongly associated with need frustration). According to this dual-process model within SDT, the lower levels of need satisfaction are not identical to the experiences of need frustration, which has a more active and undermining effect on an individual's needs (108, 109). It is thus possible that students' AIC was a strong predictor of students' positive affect, regardless of their level of need frustration.

Our findings add support to the importance of the AIC to wellbeing in traditional, collectivist societies (18). While the Bedouin culture prioritizes the values of tradition, authority, and hierarchy (52, 53) rather than values of personal autonomy and authenticity, it appears that the experience of having an AIC could be considered a wellbeing resource in this group of students. Similarly, previous research with Hong Kong Chinese youth, who is also greatly influenced by socio-cultural expectations based on tradition, hierarchy, and group orientation, found that the experience of AIC was associated with increased levels of vitality and self-esteem (17). Together, these findings underscore the SDT universality claim, which states that the satisfaction of basic needs represents essential nutrients for optimal functioning regardless of cultural differences in how autonomy is valued and prioritized (68).

Our findings should be considered under several limitations. The major limitation of the current study is a self-report bias, that might be even more pronounced in ethnic minority groups (110). The self-report method also involves problems of shared method variance, such that the associations obtained between students' need-based experiences, the sense of AIC, and their wellbeing may become artificially inflated (111). Using reports from multiple sources would be an effective way of overcoming this limitation. Another shortcoming concerns the small study sample and our limited ability to generalize our results. The Bedouin population is considered a hard-to-reach minority group (112). Like other culturally and linguistically diverse groups, multiple socio-cultural factors, including power differences (defined by situations of unequal levels of authority and influence between the researcher and research participants), reluctance to expose private issues, limited access to technology, and physical segregation (113–115), hinder Bedouin's representation in psychological and psychiatric research (116–118). Recruiting participants was also limited by the length of the spring semester during which students must face certain academic, social, and emotional challenges that we wished to investigate.

In addition to the relatively small sample size, the self-selection survey and the lack of knowledge about the website members limited our ability to obtain a random sampling (119). Because the sample consisted of students who voluntarily signed up to participate in research, it is possible that participation was confounded with various academic and psychological outcomes. Therefore, participants may not be representative of the general population of Bedouin teaching students.

Additionally, the gender distribution is biased in favor of female students. While this distribution may be representative of the general population of teaching students (74.7% female according to the 55), it does not allow us to systematically examine gender differences in the studied variables. Moreover, this study was cross-sectional and therefore cannot provide any information about causality. While need-based experiences may affect wellbeing, this link could also be reversed. It is possible, for example, that higher levels of depression and anxiety may elicit feelings of inadequacy, isolation, and pressure, which in turn will lead to more need frustration. Longitudinal designs should be employed in future research to understand the effects of AIC and need-based experiences on students' adaption along the higher-education continuum. Finally, this study investigated only students' experiences of need satisfaction or frustration, overlooking the role of the organization in supporting these needs. We encourage future research to explore how and to what degree different levels of higher education institutes (departments, lecturers, and student services) satisfy students' needs and how it is associated with students' wellbeing and academic performance.

## Conclusions

Our results show that need satisfaction and AIC act as key psychological resources in the adaptation of Bedouin teaching students to the emotional, social, and educational challenges imposed by the COVID-19. It, therefore, appears important to find ways to foster such experiences, especially in students of underserved backgrounds.

One promising venue is to adopt specific behaviors that can nurture students' needs for relatedness, competence, and autonomy. For instance, given that distance learning exacerbates Bedouin students' feelings of exclusion and social alienation (61), it would be important to find alternative ways for inclusion. Possible routes to foster experiences of relatedness would be creating working teams, initiating online social activities, and active reaching out by faculty members. Educational practices and interventions aimed at cultivating a sense of AIC and need satisfaction should consider culture-related values, norms, and expectations, including gender roles, social perceptions of mental health problems, cultural idioms of distress, and culture-specific ideas of autonomy and freedom (120, 121).

## Data Availability Statement

The raw data supporting the conclusions of this article will be made available by the authors, without undue reservation.

## Ethics Statement

The studies involving human participants were reviewed and approved by Institutional Ethics Committee, Interdisciplinary Center (IDC) Herzliya, Israel. The patients/participants provided their written informed consent to participate in this study.

## Author Contributions

RC was responsible for the conception of the study, the design, collecting data, and the statistical analyses. RC and OS contributed equally to the conceptualization, interpretations, and writing of the manuscript. Both authors contributed to the article and approved the submitted version.

## Conflict of Interest

The authors declare that the research was conducted in the absence of any commercial or financial relationships that could be construed as a potential conflict of interest.

## Publisher's Note

All claims expressed in this article are solely those of the authors and do not necessarily represent those of their affiliated organizations, or those of the publisher, the editors and the reviewers. Any product that may be evaluated in this article, or claim that may be made by its manufacturer, is not guaranteed or endorsed by the publisher.
