# Supplementary material for: An Authentic Inner Compass and Need Satisfaction as Wellbeing Resources in Bedouin Teaching Students During the COVID-19
Source: Front Psychiatry. 2022 Jul 7;13:870764. doi: 10.3389/fpsyt.2022.870764 (PMC9301382; doi:10.3389/fpsyt.2022.870764)
Supplement: Supplementary file 1 [file Table_2.DOCX]

**Appendix 1**

*Results of the confirmatory factor analysis*

|  |  |  | β | B | S.E. | C.R. | *P* |
| --- | --- | --- | --- | --- | --- | --- | --- |
| Parcel 1 | <--- | Authentic Inner compass | .98 | 3.07 | .84 | 3.65 | <.001 |
| Parcel 2 | <--- | Authentic Inner compass | .92 | .87 | .06 | 14.15 | <.001 |
| Parcel 3 | <--- | Authentic Inner compass | .40 | .32 | .08 | 3.65 | <.001 |
| Parcel 1 | <--- | Positive affect | .69 | .69 | .11 | 5.81 | <.001 |
| Parcel 2 | <--- | Positive affect | .81 | 1.38 | .23 | 5.88 | <.001 |
| Parcel 3 | <--- | Positive affect | .79 | 1.44 | .24 | 5.81 | <.001 |
| Parcel 1 | <--- | Need frustration | .77 | .90 | .14 | 6.16 | <.001 |
| Parcel 2 | <--- | Need frustration | .91 | 1.42 | .18 | 7.61 | <.001 |
| Parcel 3 | <--- | Need frustration | .69 | 1.10 | .17 | 6.16 | <.001 |
| Parcel 1 | <--- | Need satisfaction | .95 | 1.28 | .13 | 9.66 | <.001 |
| Parcel 2 | <--- | Need satisfaction | .83 | .81 | .07 | 10.35 | <.001 |
| Parcel 3 | <--- | Need satisfaction | .80 | .77 | .08 | 9.66 | <.001 |
| Depression | <--- | Psychological distress | .96 | 1.11 | .07 | 14.18 | <.001 |
| Anxiety | <--- | Psychological distress | .88 | .93 | .06 | 13.69 | <.001 |
| Stress | <--- | Psychological distress | .89 | .90 | .06 | 14.18 | <.001 |
